# Association between interleukin-10 gene rs1800896 polymorphism and diabetic retinopathy in a Chinese Han population

**DOI:** 10.1042/BSR20181382

**Published:** 2019-04-17

**Authors:** Lun Liu, Jie Zheng, Yajing Xu, Jian Gao, Lingling Fan, Dong Xu

**Affiliations:** 1 Department of Ophthalmology, The First Affiliated Hospital of Anhui Medical University, Hefei, Anhui, China; 2 Department of Ophthalmology, The Second Affiliated Hospital of Zhejiang Chinese Medical University, Hangzhou, Zhejiang, China

**Keywords:** case-control study, diabetic retinopathy, interleukin-10, single-nucleotide polymorphism

## Abstract

The levels of cytokines, inflammatory cells, and angiogenic factors increase following diabetic retinopathy (DR), and the association between interleukin-10 (IL-10) gene rs1800896 polymorphism (IL-10 -1082G/A polymorphism) and DR in different populations has been extensively studied. However, the findings are conflicting, and there is no relevant research in Chinese subjects. Therefore, this case–control study involving 327 cases (type 2 diabetes patients with proliferative DR (PDR)) and 461 controls (type 2 diabetes patients without DR) was conducted to address the relationship between IL-10 gene rs1800896 polymorphism and risk of PDR in Chinese population. Genotyping was performed using PCR-based restriction fragment length polymorphism (PCR-RFLP) assay. It was found that GG genotype or G allele of the IL-10 gene rs1800896 polymorphism was associated with decreased risk for PDR. In conclusion, IL-10 gene rs1800896 polymorphism decreases the risk of PDR.

## Introduction

Diabetic retinopathy (DR), the most common single cause of newly reported cases of blindness amongst adults [1], is a neurovascular complication of type 2 diabetes mellitus (T2DM) [2–4]. DR is characterized by injuries in neural and vascular structures [3] and is increasingly reported to be greatly influenced by inflammation [5]. The levels of cytokines, inflammatory cells, and angiogenic factors reportedly increase following DR [6]. The development and progression of DR may involve interleukin-10 (IL-10), an anti-inflammatory cytokine with potent deactivating properties.

IL-10 is expressed by most cells of the adaptive and innate immune systems, including dendritic cells, leukocytes, and macrophages [7]. IL-10 gene rs1800896 polymorphism (IL-10 -1082G/A polymorphism) in the promoter region could affect the IL-10 expression [8]. The IL-10 gene polymorphism is reportedly associated with the risk of DR in different populations [9–12]. However, this single nucleotide polymorphism (SNP) has not been investigated in Chinese populations. Therefore, we decided to investigate whether the IL-10-1082G/A polymorphism was associated with proliferative DR (PDR) in a Chinese Han population with T2DM.

## Methods

### Study population

In this hospital-based case–control design, totally 327 hospitalized T2DM patients with PDR and 461 T2DM patients without PDR were selected from the First Affiliated Hospital of Anhui Medical University between March 2014 and July 2017. T2DM was diagnosed according to the American Diabetes Association guidelines [13], while PDR was diagnosed through direct funduscopic examination. The stage of PDR was determined according to the retinopathy severity scale of the Early Treatment Diabetic Retinopathy Study Research Group. The inclusion criteria were (i) no need of permanent insulin treatment within the first year of diagnosis, (ii) no previous episodes of ketoacidosis, and (iii) age at diagnosis of diabetes ≥ 30 years. People with coronary artery diseases, peripheral vascular diseases, history of any thrombotic event, acute infection, or any other ocular disorders (e.g. branch retinal venous occlusion, glaucoma, Eales disease) were excluded. The demographic, lifestyle and clinical characteristics of all subjects were collected from medical records, including age, gender, body mass index (body mass index (BMI): kg/m^2^), hypertension and dyslipidemia. This case–control study was approved by the Ethics Committee of the Hospital and performed according to *Declaration of Helsinki*. All patients provided written informed consent prior to participation.

### Genomic DNA extraction and genotyping

Blood samples were collected using vacutainer tubes and then transferred to ethylene- diamine-tetra-acetic acid (EDTA) tubes. DNA was extracted using a Biopur Mini Spin kit (Biometrix). The genotypes of IL-10 gene polymorphism were identified by PCR-based restriction fragment length polymorphism (RFLP) assay. The PCR program was as follows: initial denaturation at 95°C for 5 min; 35 cycles of denaturation at 95°C for 30 s, annealing at 58°C for 30 s, and extension at 72°C for 30 s; final extension at 72°C for 10 min. To ensure the genotyping quality, two independent investigators interpreted the images of each gel, and at least 10% of the samples were randomly selected for repeated genotyping.

### Statistical analysis

The Hardy–Weinberg equilibrium (HWE) of the SNP genotypes was accessed by the goodness-of-fit Chi-square (χ^2^) test to compare the observed and expected genotype frequencies amongst controls. Associations between demographic characteristics and IL-10 gene rs1800896 polymorphism genotypes were assessed through χ^2^ test (for categorical variables) and Student’s *t* test (for continuous variables). Associations between the IL-10 gene rs1800896 polymorphism A/G genotypes and the risk of PDR were estimated by calculating odds ratios (ORs) and 95% confidence intervals (CIs) using logistic regression analysis. All statistical analyses were performed on SAS software package 9.1.3 (SAS Institute, Cary, NC, U.S.A.) with the significance level at *P*<0.05.

## Results

### Clinical information of the study population

The characteristics of the subjects are summarized in Table 1. The cases and controls were aged 53.58 and 52.89 years on an average, respectively, and involved 50.5 and 54.1% of males, respectively, indicating the two groups were well matched in terms of age and gender (both *P*>0.05). TC, HDL, and LDL values are listed in the left column. Significant association was found in the analyses of hypertension and dyslipidemia between cases and controls, indicating hypertension and dyslipidemia are important factors for the DR development in the Chinese Han population.

**Table 1:** Clinical manifestations of PDRy in patients with type 2 diabetes

Variable	With PDR (*n*=325)	Without DR (*n*=460)	*P*
Age (years)	53.58 ± 12.81	52.89 ± 12.29	0.440
Sex			0.325
Male	165 (50.5%)	249 (54.1%)	
Female	162 (49.5%)	212 (45.9%)	
BMI (kg/m^2^)	34.98 ± 4.78	29.44 ± 5.92	<0.001
LDL (mmol/l)	3.19 ± 0.95	3.14 ± 0.97	0.534
HDL (mmol/l)	1.22 ± 0.28	1.20 ± 0.31	0.340
TC (mmol/l)	5.48 ± 1.57	5.36 ± 1.43	0.286
Hypertension			0.001
Yes	307 (93.9%)	400 (86.8%)	
No	20 (6.1%)	61 (13.2%)	
Dyslipidemia			0.047
Yes	301 (92.0%)	404 (87.6%)	
No	26 (8.0%)	57 (12.4%)	
Duration of diabetes (years)	15.52 ± 5.86	16.30 ± 7.06	0.093

### Association between IL-10 gene rs1800896 polymorphism and PDR risk

The genotype distributions of IL-10 gene rs1800896 polymorphism in the controls conformed to the HWE (Table 2). Logistic regression analyses revealed GG genotype or G allele was related to decrease the risk for PDR (Table 2). We also evaluated the effects of the SNP on PDR risk according to patient characteristics (Table 3). The association between the rs1800896 polymorphism and PDR was only observed amongst the hypertensive subjects and older subjects (≥55 years), but was independent of gender or dyslipidemia. No significant association was found between genotype and the clinical or biochemical characteristics (Table 4).

**Table 2:** Logistic regression analysis of associations between IL-10 rs1800896 polymorphism and risk of PDR

Genotype	With PDR* (*n*=325)		Without DR* (*n*=460)		OR (95% CI)	*P*
	*n*	%	*n*	%		
AA	124		146	31.7	1.00	
AG	153		224	48.7	0.80; (0.59–1.10)	0.176
GG	48		90	19.6	**0.63; (0.41–0.96)**	**0.031**
AG+GG	201		314	68.3	0.75; (0.56–1.02)	0.063
AG+AA	277		370	80.4	1.00	
GG	48		90	19.6	0.71; (0.49–1.05)	0.083
A	401		516	56.1	1.00	
G	249		404	43.9	**0.79 (0.65, 0.97)**	**0.027**

* The genotyping was successful in 325 cases and 460 controls.

Bold values are statistically significant (*P*<0.05).

**Table 3:** Stratified analyses between IL-10 rs1800896 polymorphisms and PDR

Variable	rs1800896 (with PDR/without DR)			AG vs. AA	GG vs. AA	GG+AG vs. AA	GG vs. AG+AA
	AA	AG	GG				
Sex							
Male	63/82	74/114	26/52	0.85 (0.54–1.31); 0.453	0.65 (0.37–1.16); 0.142	0.78 (0.52–-1.18); 0.247	0.72 (0.43–1.20); 0.206
Female	61/64	79/110	22/38	0.75 (0.48–1.19); 0.222	0.61 (0.32–1.14); 0.122	0.72 (0.47–1.10); 0.130	0.72 (0.41–1.27); 0.258
Hypertension							
Yes	113/121	147/200	45/78	0.79 (0.56–1.10); 0.159	**0.62 (0.40–0.97); 0.035**	0.74 (0.54–1.01); 0.061	0.71 (0.48–1.06); 0.098
No	11/25	6/24	3/12	0.57 (0.18–1.78); 0.332	0.57 (0.13–2.42); 0.445	0.57 (0.21–1.57); 0.276	0.72 (0.18–2.87); 0.642
Dyslipidemia							
Yes	112/128	142/196	45/79	0.83 (0.59–1.16); 0.267	0.65 (0.42–1.02); 0.059	0.78 (0.57–1.06); 0.116	0.73 (0.49–1.09); 0.119
No	12/18	11/28	3/11	0.59 (0.22–1.62); 0.305	0.41 (0.09–1.78); 0.234	0.54 (0.21–1.40); 0.203	0.55 (0.14–2.15); 0.386
Age (years)							
<55	61/84	86/122	27/54	0.97 (0.63–1.49); 0.892	0.69 (0.39–1.22); 0.198	0.88 (0.59–1.33); 0.552	0.70 (0.42–1.17); 0.170
≥55	63/62	67/102	21/36	0.65 (0.41–1.03); 0.067	0.57 (0.30–1.09); 0.090	**0.63 (0.40–0.98); 0.038**	0.74 (0.41–1.32); 0.305
BMI							
<25	3/29	3/54	1/28	0.54 (0.10, 2.83); 0.464	0.35 (0.03, 3.52); 0.369	0.47 (0.10, 2.23); 0.344	0.49 (0.06, 4.28); 0.522
≥25	121/117	150/170	47/62	0.85 (0.61, 1.19); 0.354	0.73 (0.46, 1.16); 0.182	0.82 (0.60, 1.13); 0.223	0.80 (0.53, 1.21); 0.298

*The genotyping was successful in 325 cases and 460 controls.

Bold values are statistically significant (*P*<0.05).

**Table 4:** The clinical and biochemical characteristics of *rs1800960* polymorphism amongst two groups

	With PDR (*n*=325)				Without DR (*n*=460)			
	AA (*n*=124)	AG (*n*=153)	GG (*n*=48)	*P*	AA (*n*=146)	AG (*n*=224)	GG (*n*=90)	*P*
Age (years)	55.14 ± 13.34	52.91 ± 12.10	51.81 ± 13.65	0.207	52.37 ± 12.17	53.19 ± 12.45	52.87 ± 12.21	0.823
BMI (kg/m^2^)	34.65 ± 4.77	35.03 ± 4.78	35.78 ± 4.86	0.381	30.24 ± 6.24	29.16 ± 5.60	28.70 ± 5.93	0.101
TC (mmol/l)	5.57 ± 1.72	5.51 ± 1.48	5.21 ± 1.45	0.400	5.27 ± 1.42	5.44 ± 1.43	5.32 ± 1.45	0.525
HDL (mmol/l)	1.22 ± 0.27	1.21 ± 0.28	1.23 ± 0.31	0.951	1.20 ± 0.29	1.21 ± 0.31	1.19 ± 0.32	0.935
LDL (mmol/l)	3.19 ± 0.92	3.21 ± 1.00	3.06 ± 0.88	0.643	3.13 ± 0.97	3.12 ± 0.97	3.24 ± 1.01	0.611

## Discussion

This case–control study showed that IL-10 gene rs1800896 polymorphism was related to decreased risk for PDR in a Chinese population.

DR is proposed to be a manifestation of a persistent low-grade inflammation [14]. IL-10 can inhibit the production of pro-inflammatory cytokines and stimulate differentiation, proliferation and survival of some immune cells [7]. The IL-10 deficiency may not elicit protective immune response, but excessive production can exaggerate inflammatory response, resulting in immunopathology and tissue damage.

Recently, several studies explored the association between IL-10 gene rs1800896 polymorphism and DR risk. A Caucasian study first uncovered an association between this SNP and the PDR development in T2DM and demonstrated that the **GG genotype** of this polymorphism was associated with increased risk of PDR [12]. Later, a study from India investigating the relationship between this SNP and PDR risk found **GG genotype or G allele** of this polymorphism was associated with increased risk for PDR [10]. Two Brazilian studies yielded conflicting findings, as Rodrigues et al. [11] did not obtain an association, while da Silva Pereira et al. [9] found rs1800896 polymorphism increased the risk of non-PDR (NPDR), but not in PDR. Moreover, **AA genotype** of the rs1800896 polymorphism was independently associated with increased risk of NPDR, but the GG genotype was not associated with increased risk of PDR [9]. Thus, what reasons could explicate the conflicting findings? The first reason was clinical heterogeneity, since the study subjects were DR patients [11] or NPDR or PDR patients [9]. We assumed different types of DR showed genetic heterogeneity. Second, the discrepancy may be explained by different living environments and lifestyles, although they were both Brazilians. Third, the limited sample sizes [11] might not have sufficient power to reach a convincing conclusion compared with other studies.

The present study showed GG genotype or G allele carriers were related to decreased risk for PDR. Different genetic backgrounds, living environments, sample sizes, exposure factors, and clinical phenotypes of PDR may account for conflicting results of the above studies. The stratified analyses showed the risk of PDR conferred by the IL-10 gene rs1800896 polymorphism remained significant in the hypertensive and older subgroups (age ≥ 55 years), which was because susceptible individuals are likely to expose to risk factors. However, the results should be interpreted with caution because of the limited sample sizes in the stratified analyses and the limited power. Additionally, no significant association between genotype and the clinical or biochemical characteristics was observed in the present study. Nevertheless, our findings still provide evidence for a possible interaction between the rs1800896 polymorphism and some PDR risk factors.

Potential limitations of the present study should be considered. First, we only investigated PDR, but not NPDR. Second, our results were based on unadjusted estimates for confounding factors. Third, the sample size was not large enough, which might underpower our work. Fourth, no details about DR severity or treatment response were obtained, which restricted our analyses. Fifth, the underlying mechanisms of this SNP in DR should also be investigated. Sixth, multiple comparisons included in the analyses (five genetic models, and stratification of four demographic and clinical factors) may result in some false positive associations. Finally, true significance of the association between this SNP and PDR risk should be supported by further studies in different populations.

In conclusion, this case–control study indicates that IL-10 gene rs1800896 polymorphism is associated with a decreased risk of PDR. Nevertheless, this finding should be verified by further multicenter well-designed studies with larger sample sizes that include gene–environment interaction assessment.

## Abbreviations

DR: diabetic retinopathy
HDL: High-density lipoprotein
HWE: Hardy–Weinberg equilibrium
IL-10: interleukin-10
LDL: Low-density lipoprotein
PDR: proliferative DR
NPDR: non-PDR
SNP: single nucleotide polymorphism
TC: total cholesterol
T2DM: type 2 diabetes mellitus
